# Design and Development of a Wearable Device for Heat Stroke Detection

**DOI:** 10.3390/s18010017

**Published:** 2017-12-22

**Authors:** Sheng-Tao Chen, Shih-Sung Lin, Chien-Wu Lan, Hao-Yen Hsu

**Affiliations:** Department of Electrical and Electronic Engineering, Chung Cheng Institute of Technology, National Defense University No. 75, Shiyuan Rd., Daxi District, Tauyuan City 33551, Taiwan; iiccanffly@gmail.com (S.-T.C.); cwlan@ndu.edu.tw (C.-W.L.); shihaoyen@gmail.com (H.-Y.H.)

**Keywords:** wearable devices, heat stroke, biosensor, fuzzy theory

## Abstract

Heat stroke can be potentially damaging for people while exercising in hot environments. To prevent this dangerous situation, we designed a wearable heat-stroke-detection device (WHDD) with early notification ability. First, we used several physical sensors, such as galvanic skin response (GSR), heart beat, and body temperature, to acquire medical data from exercising people. In addition, we designed risk evaluation functional components that were based on fuzzy theory to detect the features of heat stroke for users. If a dangerous situation is detected, then the device will activate the alert function to remind the user to respond adequately to avoid heat stroke.

## 1. Introduction

Taiwan Island is located at the junction of the Tropic of Cancer. The northern region belongs to the subtropical zone and the southern region belongs to the tropical climate zone; both have a hot and humid climate. Influenced by global warming, the heat wave frequency and intensity are increasing. Many diseases are caused by the heat or high temperature, such as heat cramps, heat exhaustion, and heat stroke. High temperatures without wind usually cause the most serious heat stroke. For long-term exposure to hot environment while training, our military is the most high risk group. When the body’s internal heat is generated faster than the rate of heat exclusion, then the result is exertion heat illness (EHI) [1]. Outdoor runners are also exposed to this high-risk environment. Moreover, the mortality rate of heat stroke can be up to 70%, which is higher than some other diseases. Because of this, detecting and preventing heat stroke is worth exploring, researching, and discussing.

In the study of heat stroke prevention, Mizota et al., [2] suggested that heat stroke warnings could be sent to a user on their smart phone, according to the external ambient temperature-humidity data. Many studies [3,4,5] pointed out that the risk of heat stroke in high temperature is different for those of different ages. Monitoring physiological changes could be performed by using a wearable device combined with several biosensors [6,7]. However, no specific solution exists to remind users to avoid the conditions that may cause heat stroke; whereas, wearable devices are an easy and convenient solution to obtain physiological information and feedback information about the user’s body and health statistics.

Wearable devices have been widely used to change human’s lives. We can automatically and instantly monitor a runner’s physical information by wearing a device that integrates physiological sensor information. These physiological and environmental data are then used to predict the risk of heat stroke, and provides the user with the necessary action to take. The general wearable device is only limited by the ability to calculate. When biosensors receive data, the Bluetooth wireless communication is used to transmit the data to the smart phone for computing and analysis [8]. Bluetooth is embedded in all the current smart hand-held devices, having low power consumption, and being easy to connect to any smart device [9]. Although the official claims are that its transmission range is up to 100 meters, in fact, Bluetooth only transmits 5 to 10 m [10], which meets most demands of the device. However, if we want to immediately monitor the physical information from an outdoor runner, then the sensors and smart handheld device connection distance will be limited. For outdoor sports runners, if you can increase the transmission distance of the wearable device, the ease and convenience of exercise can be increased. LoRa is a suitable solution. LoRa is long range, low power, and low-throughput communication [10], making it ideal to use as a communication module for wearable devices. Notably, the wearable devices that are sold by businesses still use the Bluetooth communication as the main communication method [11]. Therefore, this paper applies the wearable device combined with LoRa wireless communication to the outdoor road running scene, aiming to improve upon the shortfall of Bluetooth communication.

In summary, wearable devices are mainly used for recording the number of exercise steps, distance, path, speed, time, and calories, etc. [11], or monitoring heart rate [8] and measuring the ambient temperature and humidity [12]. They lack early warnings for heat stroke to prevent the user from serious injury while exercising. Heat stroke warnings in wearable devices will enable users who are in a hazardous environment or in poor health be more aware of the risk of heat stroke. To address the hazard of heat stroke for runners, we designed and implemented a wearable heat stroke detection device. The major contributions of this paper include a control method via fuzzy controller for a wearable device and LoRa wireless communication. The device that we proposed can be worn on runners who are engaged in outdoor sports. The user’s heat stroke risk level was calculated using physiological and environmental information collected by the sensor, to warn the user to check their physical state to avoid the occurrence of thermal damage. The device was designed for ease of use of the system easier and to help runners avoid heat stroke.

This paper is organized as follows. In Section 2, the proposed architecture of the device is detailed. The fuzzy control strategies are illustrated in Section 3. In Section 4, an experimental verification is provided. Finally, discussion and conclusions are provided in Section 5 and Section 6, respectively.

## 2. Device Archeitecture

**Figure 1:**
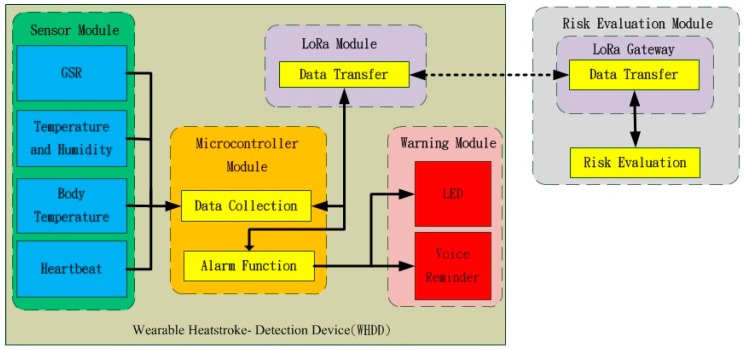
The architecture of the wearable heatstroke detection device (WHDD).

This section discusses the design of the wearable heat-stroke-detection device (WHDD) and how it was implemented. This device integrates several modules. Figure 1 describes the architecture of the system. It is divided into sensing modules, microcontrollers, LoRa wireless communication modules, a risk evaluation module, and a warning module. A three-dimensional (3D) printer was used to make the hardware container to combine all of the components. The sensors collect the environment and physiological information, and then the microcontroller performs data collection and pre-processing, and transmits the data using LoRa wireless communication to the end device to calculate the current heat stroke risk level using fuzzy logic inference. When the end device received the risk level, it sends back the recommendations to the WHDD user regarding any necessary precautions.

**Figure 2:**
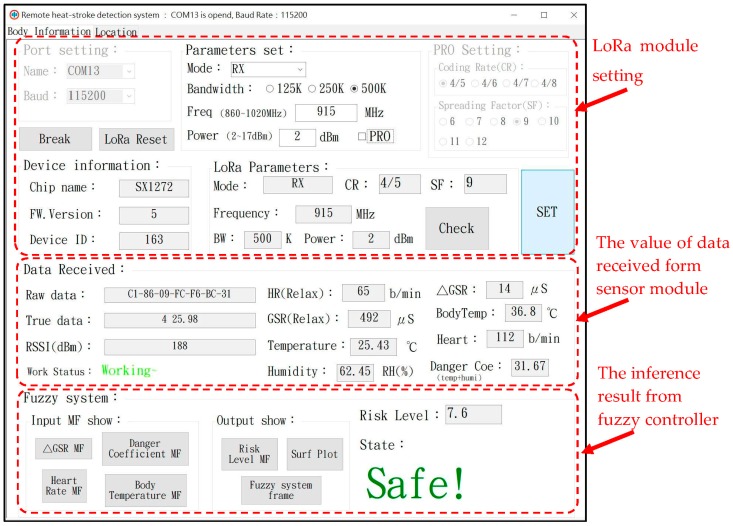
User interface of the risk evaluation module.

Figure 2 shows the user interface of the risk evaluation module. The interface was mainly divided into three parts: LoRa wireless communication parameter settings, the received physiological information, and the risk level inferred by fuzzy controller. We set the suitable LoRa communication parameters through the user interface to allow the wearable devices to obtain information and send it back to the terminal for analysis. The user interface shows the user’s risk level of heat stroke.

**Figure 3:**
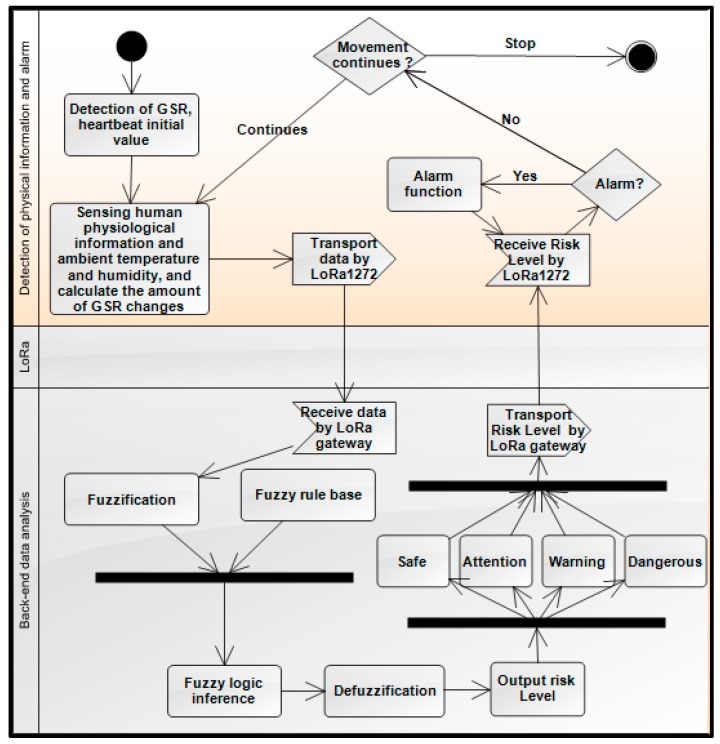
WHDD system algorithm.

Figure 3 shows the system algorithm using unified modeling language (UML). The system that is proposed in this paper aims to address the heat stroke detection and prevention methods and processes. First, the system measures the initial galvanic skin response (GSR) value and heart rate, and then begins to measure the runner’s physiological information using the IL-LoRa 1272 module to send data to the LoRa gateway. This physiological information is fed into the fuzzy controller to infer the risk of heat stroke. Finally, the alarm module will respond according to the risk level to activate to alert the user.

**Figure 4:**
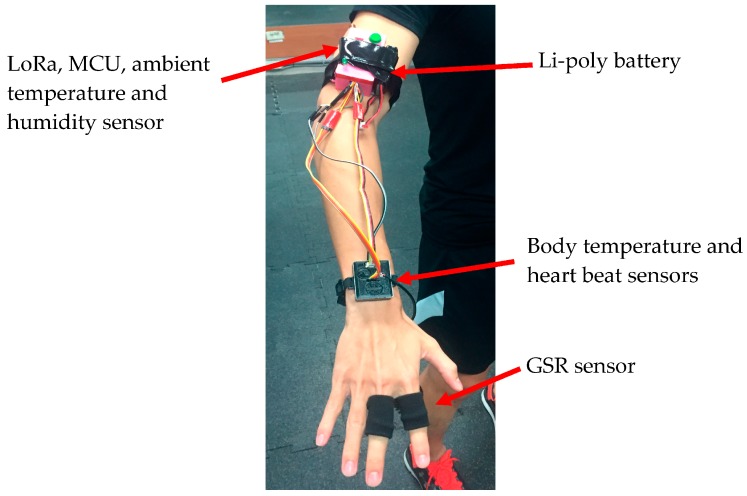
The WHDD attached to the arm of a runner.

Figure 4 shows the WHDD actual wearing condition that includes three components. The first is wearing the arm of the integrated LoRa wireless communication module, micro-controller, ambient temperature, and humidity sensor, and the alarm module device. Second is the integration of GSR, heartbeat, and the body temperature sensor worn on the ulnar head [12], which is the best position for human physiological information measurement. Adequate soft tissue is present in this position, for both position size and radius, to allow for non-intrusive photo plethysmography (PPG) measurements to successfully measure heartbeat and body temperature. The last part is power; we used a 7.4 V Li-poly battery mounted on the arm.

**Figure 5:**
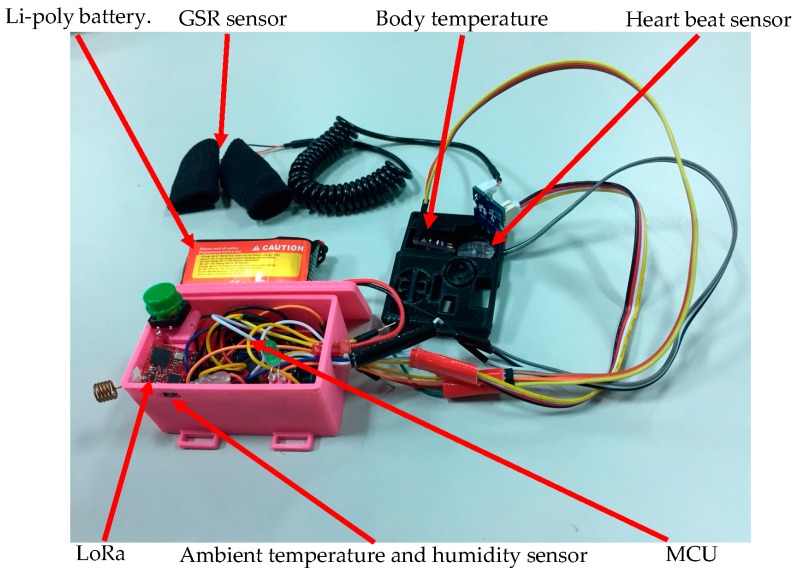
The configuration of individual sensors and components in the WHDD.

Figure 5 represents the configuration of individual sensors and components in the WHDD. We used a 3D-printer to create our WHDD case to integrate the sensor used in the system. The case makes our system more stable and sensing more accurate while the user is exercising.

### 2.1. Sensor Module

In this section, we introduce the sensors that were used in our WHDD. The GSR sensor measures the skin resistance of the human body, MLX90614 measures the body temperature changes, the pulse sensor measures the human pulse, and SHT75 measures environmental temperature and humidity.

#### 2.1.1. Galvanic Skin Response

The GSR sensor [13] measures changes in the surface resistance of the skin by releasing a micro current to the human body and amplifies the faint analog signal using the op amp (LM324PW), whereas the skin resistance (G) depends on the skin humidity, vasoconstriction and relaxation, the thickness of the stratum corneum, and chemical substances. So, when a person’s mood changes or they feel discomfort, then the skin resistance value decreases. Therefore, we used the rate of change of GSR as the evaluation index for heat stroke.

In this paper, we set the GSR sensor on the forefinger and middle finger to measure the GSR signal from runners. To calculate the change in the skin resistance value, we first estimated the runner’s GSR initial value, but this highly variable signal usually has considerable error. So, we used Equation (1) to set the system to 1 min for sampling to calculate the mean value of the GSR ($G_{base}$) as a reference for calculating the change in skin resistance. Equation (2) shows that after the system calculates the change in skin resistance (Δ*G*), this will be an indicator for estimating heat stroke risk [14,15,16,17].

(1) $$G_{base}=\frac{\sum_{0}^{N}G_{i}}{N}$$

(2) $$\Delta G=\left|G-G_{base}\right|$$

where $G_{base}$ is the initial value automatically calculated by the system, *N* is the sampling interval, which is equal to 60 s, and $\sum_{0}^{N}G_{i}$ is the total GSR value sampling in 1 min. Finally, Δ*G* is the change value in skin resistance.

#### 2.1.2. Body Temperature

During exercise, the body temperature is not only one of the most important physiological indicators, but indicators can also be used to determine the signs of heat and heat stroke. A previous study demonstrated that if the body temperature reaches 40 °C, heat stroke may occur [18].

MLX90614 [19] is a small, non-invasive sensor, with power consumption, small error characteristics, with a temperature sensing range is from −40 °C to 125 °C, with an error of ±0.5 °C. However, the temperature of the sensor is the skin surface temperature ($T_{skin}$). We used a previous process [20] to apply the core temperature estimation method of the wearable device to estimate core temperature ($T_{core}$):

(3) $$T_{core}=T_{skin}+\alpha \times \left(T_{skin}-T_{ambient}\right)$$

The body temperature of the various parts of the skin is not the same. To calculate the core temperature from the skin temperature, the temperature sensor was placed in the location where it can accurately read the core temperature estimation. The best position for human physiological information measurement is on the ulnar head, using a non-invasive sensor [12]. Moreover, our device was separated in two parts: the sensor module that is worn on the wrist and the microcontroller is worn on arm. Therefore, we used the parameter α = 0.7665 to correspond to the previous work [12], and the necessary parts of hand were near our sensor module device. The parameter α is shown in Table 1. We used the hand value of 0.7665 as the adjustment parameter.

#### 2.1.3. Heart Beat

For those who engage in outdoor sports, the heart rate monitoring can be used to observe the body’s current exercise intensity adaptation. We used a heartbeat sensor [21] to detect the human heartbeat, and for early detection of heat stroke that is caused by abnormal heartbeat.

The adult heartbeat (*H*) is usually between 60 and 100 beats per minute. Many different opinions exist about the appropriate maximum heart beat ($H_{max}$). Gulati et al., [22] used age, gender, and other physical related factors:

(4) $$H_{max}=206.3-0.711\times age$$

When exercising, the exercising heart beat ($H_{sport}$) is between 60 and 90% of the maximum heart beat. This can be used to develop effective heat stroke risk estimate indicators.

#### 2.1.4. Ambient Temperature and Humidity

We used the SHT75 sensor to detect the ambient temperature and humidity. This sensor has many advantages for developing wearable devices, such as low power consumption, small measurement error range, strong digital output signal, small size, and automatic correction function. The temperature measurement range is −40 °C to 123.8 °C, with an error value of ±0.3 °C, and the humidity measurement range is 0 to 100% RH, with an error of ±1.8% RH [23].

To more effectively use the ambient temperature and humidity data for the detection of heat stroke indicators, we referred to the Taiwan military training standards [24], as they are usually active in high temperature and humidity in the outdoor environment. Equation (5) was used to calculate the heat stroke risk factor (*R*):

(5) $$R_{\left(ET,RH\right)}=AT+RH\times 0.1$$

where $R_{\left(AT,RH\right)}$ is the thermal heat stroke risk coefficient, AT is the ambient temperature (°C), and RH is the relative humidity (%). After the calculation, the environmental risk coefficient is divided into four grades, as shown in Table 2, as an estimation of outdoor sports environmental risk indicators.

### 2.2. Microcontroller Module

The microcontroller module is the core of WHDD. The Arduino Nano board has been widely used in the field with open source code, simple development, compatibility, high volume, and small features. After the sensor detects the environment and physiological information, the microcontroller aggregates information and processes the data. Then, the information is transmitted to the risk evaluation module, and fuzzy logic inference is used to calculate the heat stroke risk level. In accordance with the evaluation results, the alarm module is controlled, feeding information back to the runners.

### 2.3. LoRa Module

**Figure 6:**
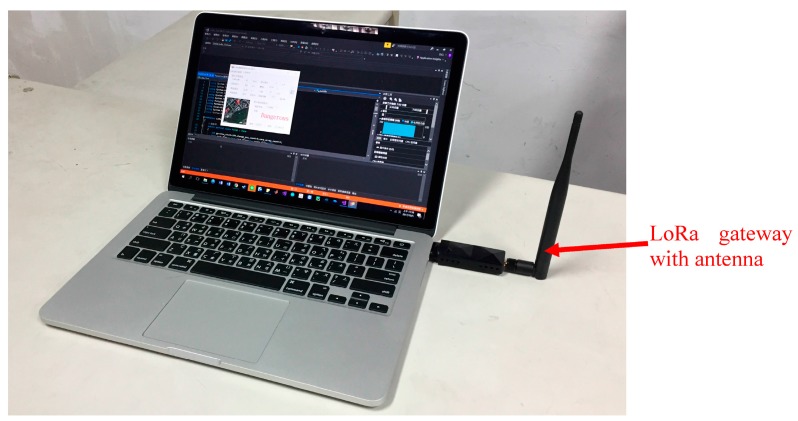
LoRa gateway module connecting to a computer.

LoRa communication has the advantages of long distance transmission, low cost, low power consumption, and having anti-jamming technology, which is suitable for use on wearable devices with low power consumption. The wearable device that is developed in this paper uses IL-LoRa 1272 [25] to transmit the sensor data. The back end receives the data obtained by the sensor via the LoRa gateway and sends a thermal heat stroke risk level to the wearable device. Figure 6 is the LoRa gateway and computer connection creating the back end device. The LoRa module is an important device in our system. This communication can reduce the power consumption and increase the data transmission distance, making it suitable for the wearable device sending information to the terminal device.

### 2.4. Risk Evaluation Module

The main function of the WHDD is building a system to automatically determine the risk level of heat stroke. The physiological data from the microcontroller is entered into and processed by MATLAB. The fuzzy logic inference processing is divided into four parts: fuzzifier, fuzzy rules, inference engine, and defuzzifier. The fuzzifier is the fuzzy controller converts the four input signals to fuzzy sets. Fuzzy rules establishment is when the fuzzy rules are created using the IF-THEN rule. Inference engine is combining the fuzzy sets and IF-THEN rules to generate fuzzy inference engine. The defuzzifier converts the inference results to the definite values.

**Figure 7:**
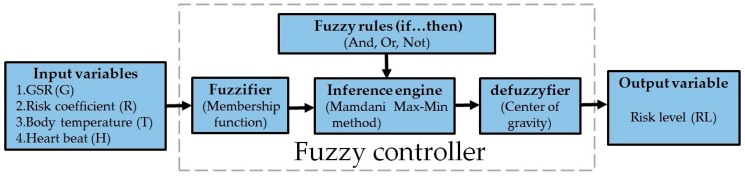
Fuzzy controller implementation process.

Figure 7 shows a fuzzy controller that is used to achieve the risk evaluation function that immediately informs the user of the risk of heat stroke. The MT2502A microcontroller collects the physiological information, including skin resistance (*G*), risk coefficient (*R*), body temperature (*T*), and heart beat (*H*) as input variables. Then, using the inference engine for fuzzy logic and using anti-fuzzy to automatically assess the user’s immediate risk level (RL). The risk level is divided into four levels: safe, attention, warning, and prohibition (Table 3), and its membership function is triangular.

In the input value, the skin resistance parameters are divided into two fuzzy sets (set), using trapezoidal and S-shaped membership function to fuzzifier. The danger coefficient from 29 to 38 was divided into four fuzzy sets, the body temperature from 36 to 45 °C was divided into three fuzzy sets, heartbeat from 60 to 220 beats per minute was divided into three fuzzy sets, and the above three input parameters were used in a π-type membership function.

The fuzzy rule was based on the subjective rule of thumb. According to the value of each input variable, the rule base was written with the if-then plus And, Or, Not logic, and then the rule base was introduced into the Mamdani fuzzy model of Max-Min Oeration to perform the fuzzy logic inference. Finally, using the center of gravity method to defuzzyfier and obtain a quantitative output value to determine the risk level.

### 2.5. Warning Device

After the completion of the risk evaluation, according the risk level, the control buzzer warns the runner, as follows: Safe—no alert, Attention—LED turns on without buzzer, Warning—LED blinks and buzzer beeps smoothly, and Prohibition—LED blinks and buzzer beeps rapidly. The alert system reminds the runner to take some precaution in response to the current situation to prevent from heat stroke.

## 3. Fuzzy Controller

In this paper, the fuzzy control system for detecting the heat stroke automatically and instantly judges the heat stroke risk level. We performed the fuzzy operation on the physiological information that was received by the microcontroller to generate the quantitative index of the final risk assessment.

### 3.1. Input and Output Variables Fuzzifier

**Figure 8:**
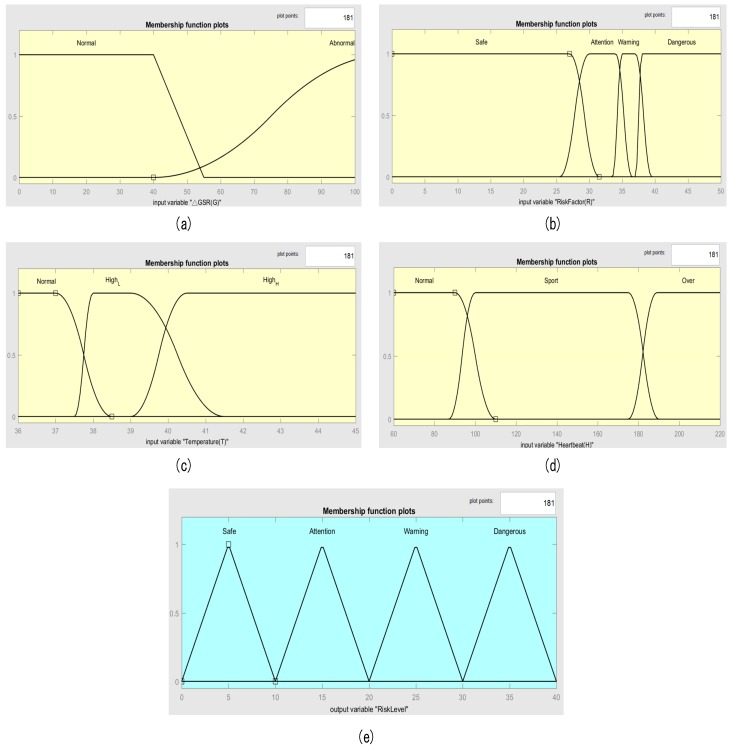
Membership function plot. (**a**) Galvanic skin response (GSR), (**b**) risk coefficient, (**c**) body temperature, (**d**) heartbeat, and (**e**) risk level.

Before performing the fuzzy logic calculation, we first confirmed the membership function to be applied to the input and output variables and set the range. Figure 8 shows the membership function graphs according to the input and output variable fuzzy sets. According to our input and output value definition (Table 3), we used the trapezoid and S-type membership function for GSR, Pi-type membership function for the risk coefficient, body temperature, and heartbeat. For output risk level, we used the triangle membership function. These membership functions are used to allow the input and output value to map between zero to one, which is an important part for the fuzzy controller.

### 3.2. Fuzzy Rules Establishment

**Figure 9:**
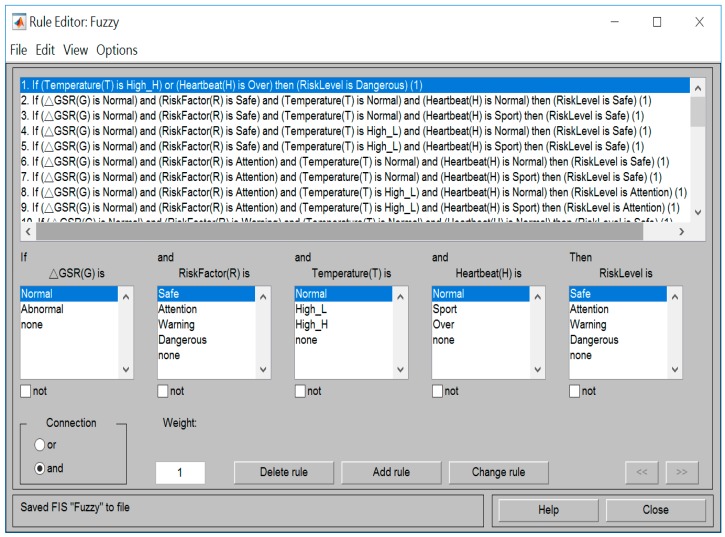
Fuzzy rule base.

The programming of the rule based in the fuzzy system is complicated, and paying attention to each value and order in the regular array is critical. If the wrong value is inputted, then the system may output the wrong risk assessment. In this paper, we used MATLAB software to build a rule base, because MATLAB provides a graphical editor of the rules. When the range of inputs and outputs has been defined and each set has been named, we could quickly develop or modify the fuzzy controller and reduce the possibility of errors. According to the name of each variable set, Figure 9 describes the 31 rules that we established in this paper. These rules will influence the fuzzy controller to make a decision by inference engine.

### 3.3. Fuzzy Logic Inference

After accomplishing all of the variable fuzzy sets and establishing the rules, we used MATLAB to calculate the fuzzy logic inference by manually entering each variable value to obtain a quantitative indicator that we called the risk level of heat stroke.

**Figure 10:**
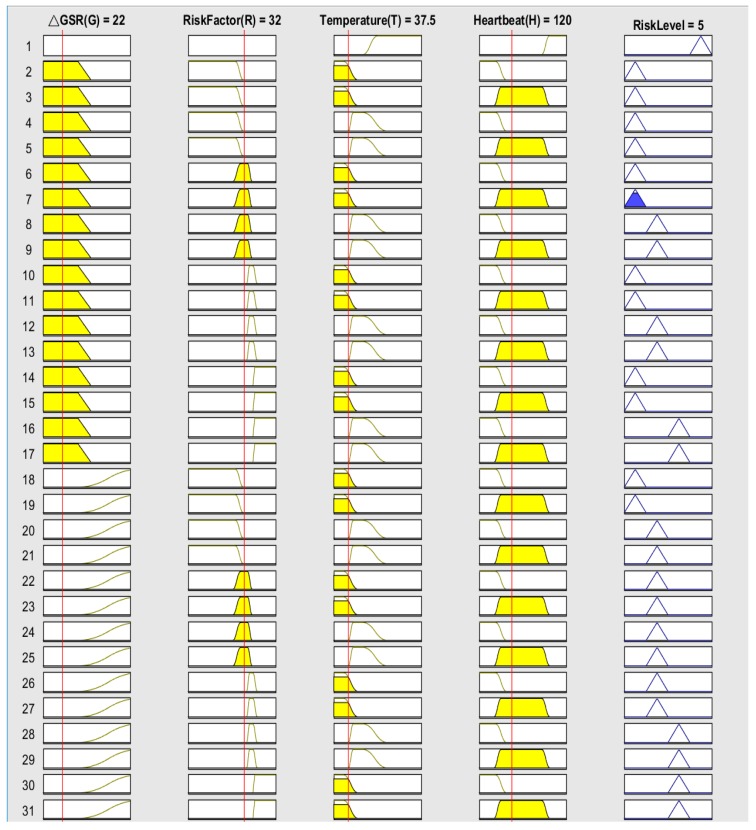
The risk level of heat stroke corresponded to safe status.

For example, four inputs were entered into the fuzzy controller. We set the variable [ΔGSR(*G*), Risk factor (R), Body temperature (T), Heart beat (H)] = [22, 32, 37.5, 120] for input data that were entered into the fuzzy controller to calculate. The obtained results are shown in Figure 10. The fuzzy controller used the 31 fuzzy rules that we established to infer the output risk level from four input values. The risk value was five, which corresponds to “Safe” status, and the device will not send anything back to the terminal to alert the runner.

## 4. Experiment

To verify the WHDD, we designed an experiment for the treadmill for testing the monitoring of the physiological information of the runner.

### 4.1. Experiment Design

**Figure 11:**
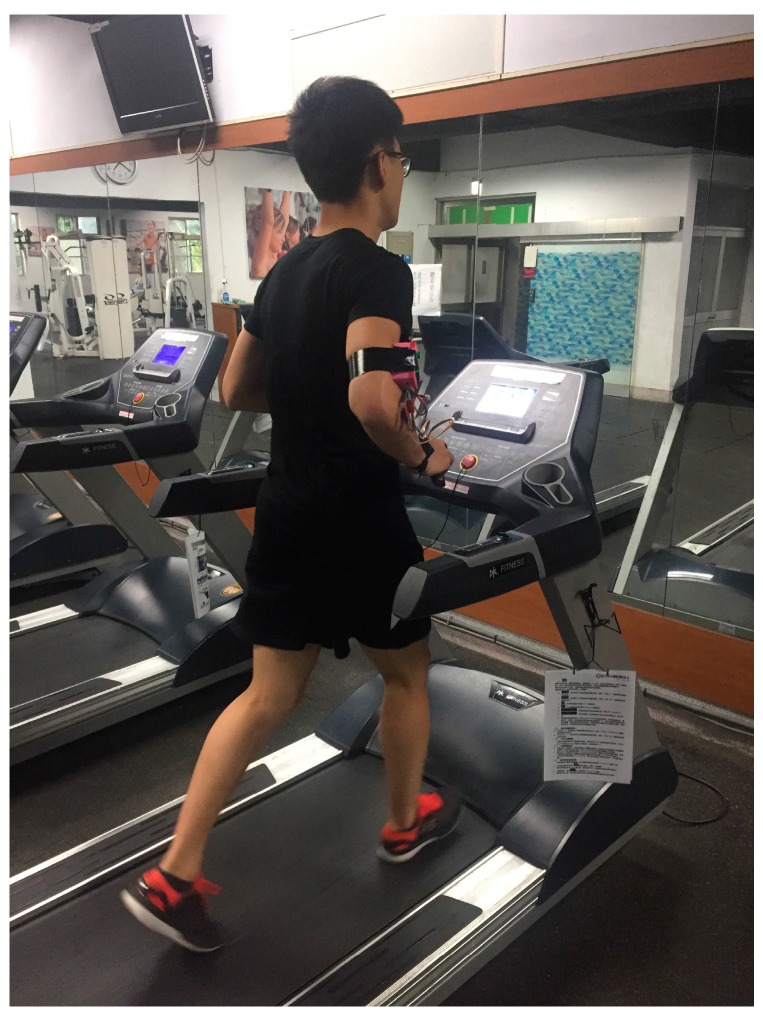
Experiment environment.

**Figure 12:**
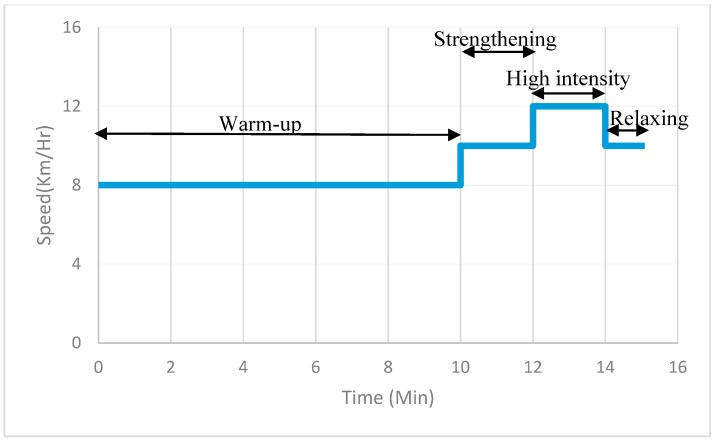
The speed changing diagram of treadmill.

A 35-year-old man with no previous exercise routine participated in this experiment. Figure 11 shows that the participant is wearing the WHDD and running on the treadmill. In the experiment process, we did not open any ventilation equipment or air conditioner so as to avoid affecting the experimental environment. Because we used ambient temperature and humidity to calculate the environmental risk coefficient and both of these variables changed slightly, we only tested the relationship between the sport intensity and two variables: heartbeat and body temperature changes. To create different sport intensities, the runner experienced four stages: warm-up, strengthening, high intensity, and relaxing. Figure 12 shows the speed setting and time configuration of the treadmill. In our experiment, we used three different speeds during a 15-min span. In the warm-up stage, the treadmill speed was 8 km per hour for 10 min. Then, the speed was increased to 10 km per hour for 2 min in the strengthening stage. After that, the treadmill speed was 12 km per hour for 2 min in the intense stage. Finally, the treadmill speed was 10 km per hour for 1 min in the relaxing stage. We used the speed change to verify and determine the effect of exercise intensity on the physiology information.

### 4.2. Experiment Results

The main purpose of this paper was to design a wearable device that automatically and instantly monitors the physical condition of a runner using biosensors, and then transmits data to the controller to calculate the risk of heat stroke using fuzzy logic inference. Then, according to the risk level, provide the user with an alert to avoid heat stroke damage.

**Figure 13:**
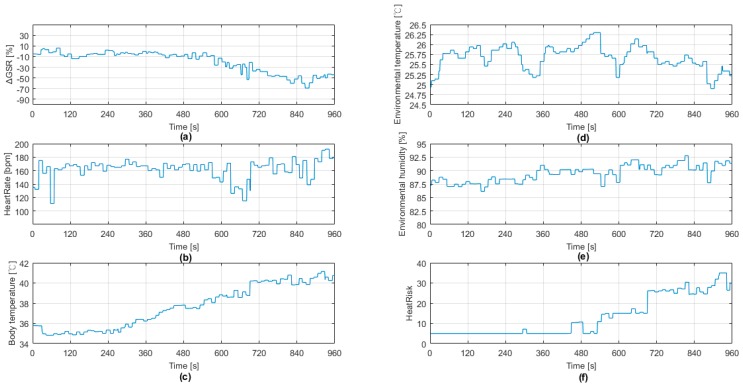
WHDD experiment results, with physiological information about (**a**) GSR, (**b**) heartbeat, (**c**) body temperature, (**d**) ambient temperature, (**e**) ambient humidity, and (**f**) the inferred risk of heat stroke provided by the evaluation system.

Figure 13 shows the results of the physical data and the heat stroke risk inference result. Figure 13a shows that with the increasing running intensity and time, GSR gradually decreased, which should be due to human sweating. Figure 13b shows the experimental human heart rate. The maximum heart rate of 192 bpm occurred at 180 s. According to the experimental design, this result matches the runner’s actual situation, just changing from the intense phase into the relaxation phase. Figure 13c shows that with the increase in running time, the body temperature gradually increased. In addition, the ambient temperature measured in Figure 13d shows a change of 1.5 °C, whereas the ambient humidity in Figure 13e shows a slight increase over time. By using these physiological data through the fuzzy logic inference, we obtained Figure 13f that shows a trend matching the four stages of our experiment design from warm-up, strengthening, high intensity, and relaxing. We determined that WHDD uses the physiological information to infer the heat stroke risk level, which could truly reflect the risk of heat stroke.

## 5. Discussion

By reviewing Figure 12 and Figure 13f, we determined that the risk of heat stroke measured by the system could react early to the runner’s speed prior to the stage transition. For example, 68 s before the warm-up stage changed to the strengthening stage, the heat stroke risk level obviously increased. The system calculated the risk level increased 117.60% at the beginning of the level. When transitioning to the strengthening stage in 600 s, the risk level increased two-fold. From the fierce to the relaxing stage, the risk level did not instantly decrease. We consider that the reason for this result is because the body needs time to cool down after exercise.

From the above analysis of experimental data, the wearable device that is designed in this paper could function as an early warning of thermal stroke. The risk value evaluated by the system mimicked the change curve of the runner during the exercise phase. Therefore, by using a wearable device to monitor the runner’s physiological information, early warnings of risk conditions to avoid the occurrence of heat stroke could be provided to the runner.

### 5.1. Detection of Heat Stroke Risk Indicator

We reviewed the prior literature for interest in wearable devices for joggers [11]; most studies focused on recording the path, distance, heartbeat, and so on. However, the research lacked an indicator to inform joggers of potentially dangerous situations in terms of heat stroke. Between the Figure 12 and Figure 13f, our WHDD successfully transformed user physiological information into a heat stroke risk indicator using fuzzy logic inference, matching well with the user’s current body experience.

### 5.2. Heat Stroke Alerting System

Heat strokes are apt to occur when people exercise in hot environments. Sometimes people are not aware when they are experiencing the early stages of heat stroke. For any disease, prevention is better than cure, especially for heat stroke. In our experimental stage, the WHDD detected the exercise intensity and alerted the user to lower the intensity of exercise or take a rest to prevent the heat stroke from occurring.

### 5.3. LoRa Wireless Communication Implementation

The device used the LoRa wireless communication technique. When compared to Bluetooth, the low power consumption makes it more suitable for the long-term use in wearable devices. LoRa also has a larger transmission range.

## 6. Conclusions

For people who live in subtropical or tropical regions, running in hot temperatures is unavoidable. This paper proposed a wearable device to ensure people can remain safe when exercising outdoors. WHDD was designed to monitor the physical information of outdoor runners, and to determine the possibility of a heat stroke occurring while running. We used several sensors to monitor physiological information through the micro-controller, including skin resistance, heart rate, and body temperature data, combined with ambient temperature and humidity. The data was sent to an end device to calculate the risk level using fuzzy logic inference. The system detected the risk level and alerted user to watch their body status to prevent heat stroke from occurring. This device could allow everyone exercising in the heat to never worry their safety and be healthier. From the results of the experiment, HWDD detected the trend in the runner’s physiological information in advance of the exercise intensity, and proving that WHDD could specifically prevent the occurrence of heat stroke.

## Figures and Tables

**Table 1:** Tuning parameters for the core temperature in each body part.

Body Part	**α**
Rectal	0.0699
Head	0.3094
Torso	0.5067
Hand	0.7665
Foot	2.1807

**Table 2:** Heat stroke risk coefficient.

Risk State	Risk Coefficient	Heat Stroke Precaution
Safe	≤29	Normal activity is OK.
Attention	30–34	Normal activity is OK, but need to drink water frequently.
Waring	35–37	Avoid the high intensive work, and need to drink water frequently.
Dangerous	≥38	Avoid the outdoor activity, and force to drink water.

**Table 3:** Inputs and output value definition.

Var	No.	Name		Value Range Definition			
In	1	G	Status	Normal	Abnormal	-	-
			Range	G ≤ 50	50 < G ≤ 100	-	-
	2	R	Status	Safe	Attention	Warning	Dangerous
			Range	R ≤ 29	30 ≤ R ≤ 34	35 ≤ R ≤ 37	R ≥ 38
	3	T	Status	Normal	Slightly higher	Too high	-
			Range	36 ≤ T ≤ 37	38 ≤ T ≤ 40	41 ≤ T ≤ 45	-
	4	H	Status	Normal	Exercise	Too high	-
			Range	60 ≤ H ≤ 99	100 ≤ H ≤ 188	189 ≤ H ≤ 220	-
Out	1	RL	Status	Safe	Attention	Warning	Prohibition
			Range	0 ≤ D ≤ 10	11 ≤ D ≤ 20	21 ≤ D ≤ 30	31 ≤ D ≤ 40
